# Visual Motion Induces a Forward Prediction of Spatial Pattern

**DOI:** 10.1016/j.cub.2011.03.031

**Published:** 2011-05-10

**Authors:** Neil W. Roach, Paul V. McGraw, Alan Johnston

**Affiliations:** 1 Visual Neuroscience Group, School of Psychology, The University of Nottingham, Nottingham NG7 2RD, UK; 2 Department of Cognitive, Perceptual and Brain Sciences and CoMPLEX, University College London, London WC1H 0AP, UK

## Abstract

Cortical motion analysis continuously encodes image velocity but might also be used to predict future patterns of sensory input along the motion path. We asked whether this predictive aspect of motion is exploited by the human visual system. Targets can be more easily detected at the leading as compared to the trailing edge of motion [1], but this effect has been attributed to a nonspecific boost in contrast gain at the leading edge, linked to motion-induced shifts in spatial position [1–4]. Here we show that the detectability of a local sinusoidal target presented at the ends of a region containing motion is phase dependent at the leading edge, but not at the trailing edge. These two observations rule out a simple gain control mechanism that modulates contrast energy and passive filtering explanations, respectively. By manipulating the relative orientation of the moving pattern and target, we demonstrate that the resulting spatial variation in detection threshold along the edge closely resembles the superposition of sensory input and an internally generated predicted signal. These findings show that motion induces a forward prediction of spatial pattern that combines with the cortical representation of the future stimulus.

## Highlights

► The human visual system uses motion information to construct a forward model ► Sensory thresholds depend on superposition of internal prediction and sensory input ► Constructive interference improves detectability of stimuli matching prediction ► Destructive interference reduces detectability of stimuli inconsistent with prediction

## Results and Discussion

**Figure 1:**
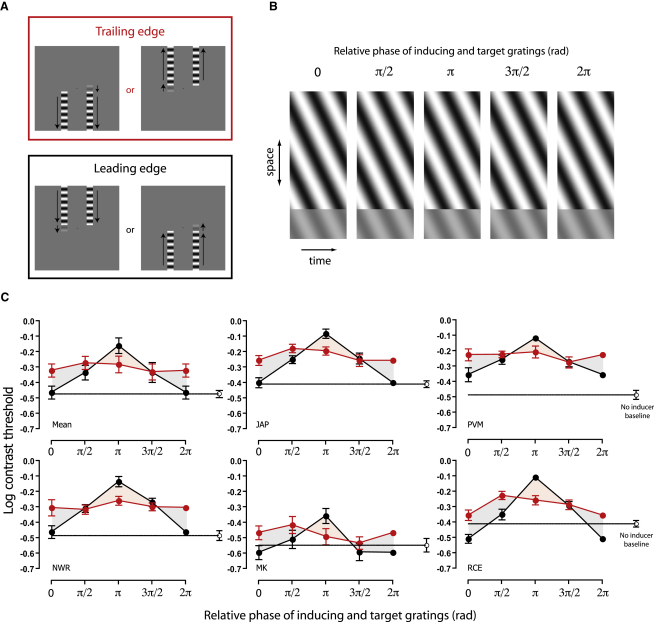
Phase-Dependent Suppression of Contrast Sensitivity at the Leading Edge of a Moving Stimulus (A) Stimulus configuration. Observers were required to indicate whether a small target pattern was presented to the left or right of fixation. Two high-contrast inducer gratings were positioned adjacent to the potential target locations. Each drifted in the same direction and at the same speed as the target. Manipulation of the stimulus configuration allowed comparison of performance when the target was positioned at either the trailing (red box) or leading (black box) edge of the inducer. (B) Space-time plots depicting different phase relationships between inducing (high-contrast region) and target (low-contrast region) stimuli. Note that in-phase(0 or 2π) targets are consistent with a continuation of the inducer waveform in space and time, albeit at a lower contrast. (C) Detection thresholds for trailing-edge (red symbols) and leading-edge (black symbols) conditions, plotted as a function of the relative phase of target and inducer stimuli. Open symbols indicate thresholds for a baseline condition with no inducers present. The mean performance of five observers is displayed in the upper left panel, with the remaining panels showing individual data. Error bars indicate ± 1 standard error, calculated either across observers (mean plots) or via bootstrapping (individual thresholds). See also Figure S1.

We measured changes in observers' ability to detect a small drifting target pattern when it was presented at the end of an inducer stimulus drifting in the same direction and at the same speed. In different conditions, we manipulated whether the target pattern was positioned at the trailing or leading edge of the inducer (see Figure 1A) as well as the relative phase of the two stimuli (see Figure 1B). As shown in Figure 1C, detection thresholds at the trailing edge were elevated relative to a baseline condition with no inducer (mean suppression = 0.17 log units or 49%). No systematic variation of performance with stimulus phase was evident, consistent with previous reports of surround suppression effects [5–7]. At the leading edge, however, we found that performance was highly phase dependent. Thresholds for target patterns that were in phase with the inducer matched baseline performance (mean suppression = 0.01 log units or 2%), whereas thresholds for antiphase test patterns were highly elevated (mean suppression = 0.31 log units or 106%). The fact that phase dependence occurs at the leading edge but not at the trailing edge means that it cannot be due to the introduction of a discontinuity in the luminance profile at the target/inducer border. Neurones with receptive field centers beyond the edge of the moving stimulus may respond to the inducer, but passive spatial filtering cannot explain the different pattern of results at the trailing and leading edges. Our results are also incompatible with any mechanism that might generally improve or impair performance, such as shifts in spatial attention [8, 9], induction of eye movements [10], or flank facilitation [11, 12]. Instead, our results suggest an anticipatory modulation of the signal-to-noise ratio ahead of moving patterns: suppression is released for targets with a form that is consistent with continued motion of the inducer along its trajectory but intensified for targets that are inconsistent.

How does the visual system accomplish such a highly specific adjustment of sensitivity? One possibility is that a predictive signal generated by a forward model representing the likely future pattern of visual input is simply added to the incoming test pattern, thereby boosting compatible signals and reducing threshold. Superposition would increase the effective contrast of targets that are in phase with the inducer (through constructive interference) and decrease the effective contrast of targets that are in antiphase (destructive interference). Note that this proposed interaction between the prediction and the signal implies they must have the same type of neural representation, making addition a permissible operation.

**Figure 2:**
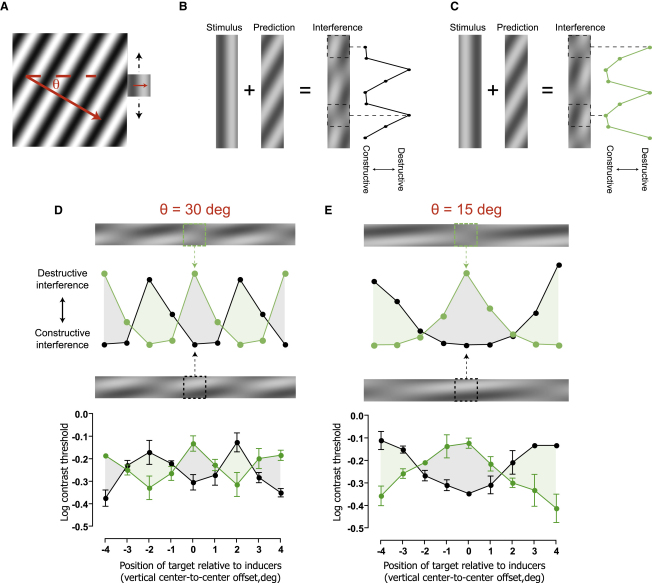
Interference between Sensory Input and Internally Generated Predictions (A) Example of a stimulus configuration in which the drift direction of a large inducer grating has been rotated by 30° relative to a small, rightward-drifting target. (B) Computation of the theoretical spatial interference profile produced by superposition of the target and a forward model of the inducer. The stimulus panel represents the target at all possible positions along the inducer's edge. Summing this with a prediction formed by extrapolating the inducer pattern results in a spatial interference profile. Locations at which target detectability is expected to benefit from constructive interference are indicated by regions of higher contrast (e.g., upper dashed box), whereas locations at which detectability is expected to be hampered by destructive interference are indicated by lower contrasts (e.g., lower dashed box). Calculating the local contrast in the interference profile at each target location allows an approximation of the expected modulation of target detectability along the edge of the inducer. (C) Shifting the starting phase of the target by half a cycle, while holding all other factors constant, produces a concomitant shift in the theoretical spatial interference profile. (D) Comparison of theoretical (upper panel) and empirical (lower panel) spatial interference profiles for an inducer rotated by 30°. Theoretical interference profiles are reproduced from (B) (black symbols) and (C) (green symbols). Mean detection thresholds for three observers are shown, plotted as a function of the target's position relative to the vertical midpoint of the inducer (see Experimental Procedures for details). (E) Comparison of theoretical (upper panel) and empirical (lower panel) spatial interference profiles for an inducer rotated by 15°. Error bars indicate ± 1 standard error. See also Figure S2.

To test this idea, we next investigated the possibility of creating more complex modulations of sensitivity by inducing predictions at a different angle to the target pattern. Figure 2A shows a stimulus configuration in which the orientation of a large drifting inducer pattern has been rotated 30° clockwise relative to that of a smaller target. In this situation, the correspondence between target and prediction will depend on the position of the target stimulus along the inducer's edge. This is illustrated in Figure 2B, which shows the spatial interference pattern produced by simply summing an extended version of the target pattern with a prediction formed by extrapolation of the inducer grating. Regions of high and low contrast indicate target positions at which interference is expected to be constructive and destructive, respectively. If the brain combines sensory input with internally generated predictions in this manner, observers' ability to detect the target should modulate with a predictable period as it is moved along the edge of the inducer. In addition, by manipulating the initial phase of the target, it should be possible to alter the phase of this space-variant modulation (see Figure 2C). As shown in Figure 2D, the experimental results clearly supported these hypotheses. Observers' thresholds modulated systematically as a function of target position, mirroring the expected interference between sensory and predicted input. To test the generality of this effect, the experiment was repeated using an inducer pattern that was rotated by 15° relative to the target. This broadened the spatial modulation, both in the theoretical interference profile and the threshold measurements (Figure 2E). Together with our initial findings, these results provide strong evidence that stimulus detectability in the region of space ahead of a moving object is determined by the sum of sensory input and a predictive signal. Moreover, the fact that interference occurs between these entities implies that the brain must treat its own predictions like real sensory signals.

**Figure 3:**
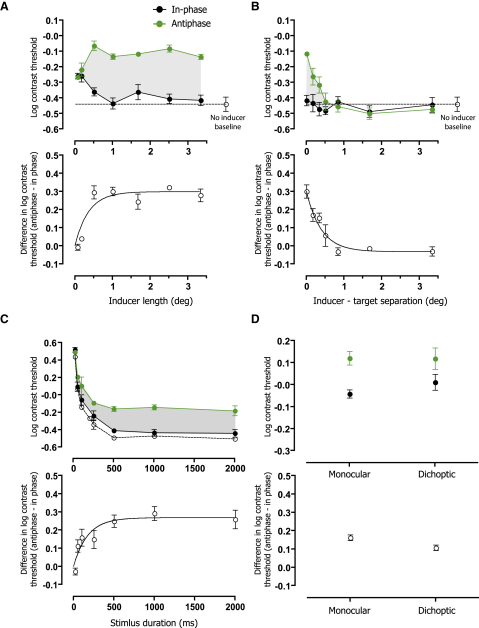
Properties of the Predictive Model (A) Spatial support. Upper panel shows mean detection thresholds of three observers for in-phase (black) and antiphase (green) targets, plotted as a function of inducer length. For comparison, the open symbol shows performance with no inducer stimuli. The growth of the interference effect is summarized in the lower panel, which shows the difference between in-phase and antiphase thresholds along with the best-fitting exponential function (semisaturation space constant = 15 arcmin). (B) Spatial projection. Thresholds are shown as a function of the size of the spatial gap introduced between target and inducer stimuli. The interference effect is restricted to a small spatial region ahead of the inducer (space constant of exponential fit = 25 arcmin). (C) Time course. Thresholds plotted as a function of the duration of target and inducer stimuli reveal the buildup of the interference effect over time (temporal semisaturation constant = 122 ms). (D) Interocular transfer. Comparison of performance in conditions where target and inducer stimuli were presented either to the same eye (monocular) or to different eyes (dichoptic) suggests that predictions formed based on motion information presented to one eye can interfere with visual input to the other. Error bars indicate ± 1 standard error.

Our approach provides a direct and versatile tool for quantifying predictive modeling in the visual system and revealing the characteristics of the underlying mechanisms. To determine the extent of the spatial region over which motion information is pooled to form a prediction, we repeated our original experiment while systematically manipulating the length of the inducer stimuli. Performance for in-phase (black symbols) and antiphase (green symbols) targets presented at the leading edge of the inducer is shown in Figure 3A. Thresholds for the two conditions rapidly diverged with increasing inducer length, before reaching an asymptotic level beyond approximately 1° of visual angle. This suggests that the predictive model is supported by computations occurring within a local region of the visual field. We also determined how far the prediction extends in space by measuring the effect of introducing a gap between inducer and target stimuli. This produced a graded attenuation of the interference effect over a similarly narrow spatial range (see Figure 3B). The local support and projection of the predictive model, coupled with its high degree of phase specificity, suggest that it is most likely implemented at a relatively early stage of visual processing where neuronal receptive fields are small and information about contrast polarity is retained [13, 14]. Manipulation of stimulus duration further revealed the dynamic nature of the predictive model (see Figure 3C). Interference was evident for stimulus presentations as brief as 50 ms and continued to grow over a period of approximately 500 ms.

To better isolate the stage of processing at which the predictive model is formed, we next investigated the effect of presenting inducer and target stimuli to different eyes. Because signals from the two eyes converge only after the input layers of primary visual cortex (V1) [13, 14], measurement of interocular transfer provides a means of assessing whether the predictive model has a cortical or subcortical locus. Figure 3D compares observers' performance when inducer and target stimuli were presented either to the same eye (monocular) or to different eyes (dichoptic). Differences between the detectability of in-phase and antiphase targets were evident in both conditions, suggesting that predictions formed on the basis of visual input to one eye can interfere with input to the other. Thus, we can be confident that generation of the predictive model must involve visual cortex. Although precise determination of the neural circuitry involved requires further investigation using neurophysiological techniques, our combined psychophysical results point toward a likely stage of processing at or soon after the output layers of V1. We also introduced retinal motion using static stimuli by having observers track a fixation cross that translated across the screen (see Figure S3 available online). For retinal motion induced by tracking, we found no differences in detection threshold for in-phase targets compared with antiphase targets at the leading edge of the inducer. This implies that prediction is based on object motion rather than retinal motion.

Motion can induce a shift in the perceived position of a Gaussian-windowed sine function [15]. However, our data allow us to rule out the possibility that the target stimulus is simply added to a spatially shifted version of the inducer, because the phase of the modulation in threshold would include this shift, whereas our interference data suggest superposition with an in-phase extension of the inducer. Several characteristics of motion-induced spatial shifts and motion-induced predictions also differ: the former increases with viewing eccentricity [15], varies nonmonotonically as a function of duration [2], and is demonstrable with contrast-defined motion [16] whereas the latter decreases with eccentricity (see Figure S4), has a monotonic relationship with duration (Figure 3C), and is absent for contrast-defined motion (see Figure S5). We can therefore distinguish between these motion-related phenomena, which would appear to have different underlying mechanisms.

Forward models have been successful in explaining how skilled action can be accomplished without the delays inherent in closed-loop feedback circuits [17, 18]. They may play a similar role in sensory systems. Discrepancies between sensory predictions and sensory input could be used as an internal error signal for recalibrating local motion detectors. This would provide the visual system with a means of maintaining the accuracy of velocity estimates and help optimize observers' ability to interact with their dynamic surroundings. Forward models of motor actions are also thought to allow the brain to cancel the reafferent sensory feedback that results from self-movement [19, 20]. In contrast to attenuating predicted sensory signals, however, our results suggest that the visual system employs forward modeling to maintain its ability to detect predictably moving objects. Constructive interference produced by the superposition of sensory signals with well-matching forward predictions acts to counteract surround suppression. This clearing of the path ahead of moving objects may contribute to the impressive sensitivity of the human visual system to predictable motion trajectories [21–23]. The magnitude of any improvements in sensitivity obtained via this mechanism may need to be tempered against the detrimental effects of destructive interference, occurring when the form of sensory input differs from internal predictions. One situation in which this problem could manifest is when an object's form changes as it moves. Indeed, it has recently been shown that motion impairs the ability of observers to perceive changes in object characteristics [24].

This study adds to a growing body of work suggesting that, rather than simply analyzing sensory information in a passive manner, the brain exploits contextual information to actively predict the nature of input it receives [25–27]. Our findings are also broadly consistent with previous suggestions that the visual system attempts to extrapolate the position of moving objects [28, 29]. What our results demonstrate for the first time is that forward predictions formed based on motion information precisely specify the pattern (i.e., phase and orientation) of expected future visual input. Moreover, we show that these internally generated predictions interfere with representations of actual visual input in a lawful manner, providing a direct and objective means of studying forward modeling in the visual system.

## Experimental Procedures

### Observers

Eleven observers aged from 21 to 41 participated in the study: two of the authors (N.W.R. and P.V.M.) plus nine individuals who were naive to the specific purpose of the experiments. All had normal or corrected-to-normal visual acuity and gave informed consent.

### Apparatus

Stimuli were presented on a gamma-corrected 100 Hz Mitsubishi Diamond Pro 2045U monitor driven by a Cambridge Research Systems ViSaGe with 14-bit resolution. The display was viewed binocularly from a distance of 65 cm, with head position maintained using a chin rest.

### Phase Dependence of Suppression at Leading and Trailing Edges

Observers (n = 5) were instructed to detect a small target grating (width = 1°, height = 1°, spatial frequency = 1 c/°) presented for 1000 ms at a location either 2° to the left or right of a small fixation cross. The target drifted upward or downward (randomly selected on each trial) with a temporal frequency of 5 Hz.

Two high-contrast inducer gratings were displayed either above or below the potential target locations and drifted in the same direction and at the same speed as the target (width = 1°, height = 6.67°, spatial frequency = 1 c/°, temporal frequency = 5 Hz, Michelson contrast = 100%). This arrangement ensured that the target abutted one of the inducer stimuli on any given trial but that the inducers provided no cue as to the location of the target. As depicted in Figure 1A, manipulation of the stimulus configuration allowed comparison of performance where the target abutted either the leading or trailing edge of the nearest inducer. Across different conditions, the waveform of the target was either in phase with the inducer (indicated by a relative phase of 0 or 2π) or advanced by one-quarter (π/2), half (π), or three-quarters (3π/2) of a cycle. Figure 1B shows space-time profiles of a downward-drifting target stimulus (low-contrast region) presented at the leading edge of an inducer positioned above it (high-contrast region). Performance was also assessed in a baseline condition with no inducer stimuli. To avoid the introduction of spatial and/or temporal uncertainty, we used thin (2 arcmin) line cues to mimic the vertical edges of the pair of inducers present in other conditions.

Target detectability was measured using the method of constant stimuli, with 120 trial presentations for each of seven equally log-spaced Michelson contrast levels (840 trials contributing to each threshold estimate). Individual psychometric functions were fitted with a logistic function, and contrast detection thresholds were defined as the Michelson contrast (expressed as a percentage) yielding 75% correct performance. Trials for leading-edge and trailing-edge conditions were randomly interleaved in blocks with a fixed phase difference.

### Spatial Interference Profiles

An example of the stimulus configuration used to measure spatial interference patterns is shown in Figure S2. The target (0.5 c/°, 5 Hz, 2° × 2°) was presented at an eccentricity of 4° to the left or right of a small fixation cross. The target was vertically oriented and drifted toward fixation (i.e., rightward when presented on the left and vice versa). The directions of the two inducer gratings (width = 10°, height = 10°, spatial frequency 0.5 c/°, temporal frequency = 5 Hz, Michelson contrast = 100%) were rotated by either 30° or 15° with respect to that of each potential target (indicated by θ in Figure 2A). Across different blocks of trials, the relative position of target and inducer was controlled by vertically displacing the inducers (target positions remained constant). In the example shown in Figure S1, the inducers were displaced upward by 4°, corresponding to a center-to-center offset of +4°, as displayed in Figures 2C and 2D. Conditions with targets differing in phase by half a cycle were interleaved within each block. Contrast detection thresholds were estimated using methods identical to those described above for three observers.

### Properties of the Predictive Model

Methods were comparable to those described above for the phase-dependence experiment, with the exception that target stimuli were always presented at the leading edge of the inducer. In-phase and antiphase target conditions were randomly interleaved within each run of trials.

#### Spatial Support

The length of the inducer stimuli was varied between 0.067° (4 arcmin) and 3.33°.

#### Spatial Projection

Inducer stimuli (length = 6.67°) were shifted vertically to introduce a spatial gap between inducer and target of between 0.067° (4 arcmin) and 3.33°. Note that the phase of the inducer stimuli was adjusted to accommodate the gap, such that for in-phase conditions, the form of the targets remained consistent with extrapolation of the inducer in space and time.

#### Time Course

The duration of all stimuli (target and inducer) was manipulated between 20 ms and 2000 ms.

#### Interocular Transfer

Stimuli for the two eyes were presented in different regions of a single monitor and viewed using a mirror stereoscope. The viewing field for each eye was framed by a rectangular peripheral fusion lock. In monocular conditions, both target and inducer stimuli were presented to the same eye, which was randomly determined on each trial. In dichoptic conditions, the target was presented to one eye (randomly determined) while the inducer stimuli were presented to the other.

## Supplemental Information

Document S1. Five Figures
