# Supplementary material for: Visual Motion Induces a Forward Prediction of Spatial Pattern
Source: Curr Biol. 2011 May 10;21(9):740–5. doi: 10.1016/j.cub.2011.03.031 (PMC3093611; doi:10.1016/j.cub.2011.03.031)
Supplement: Document S1. Five Figures [file mmc1.pdf]

Current Biology, Volume *21*

Supplemental Information

Visual Motion Induces a Forward Prediction of Spatial Pattern

Neil W. Roach, Paul V. McGraw, and Alan Johnston

Supplemental Results

Dissociating Retinal and Object Motion

To this point our experiments all employed drifting stimuli viewed under steady fixation. To dissociate retinal and object motion, we presented static inducer and target stimuli and induced retinal motion by having observers track a moving fixation cross (see Figure S2A). Stimulus parameters were as described above for the original phase-dependency experiment. At the beginning of each trial, a cross appeared at a location 5° above or below the centre of the screen. After a 1000 ms delay, the cross moved vertically at 5°/s for 1000 ms. Tracking of the cross induced retinal motion of inducer and target stimuli in the opposite direction. Figure S2B shows that no differences in contrast threshold were found between in-phase and antiphase targets presented at the leading edge of the inducer.

To test the generality of this effect we also repeated the experiment while (1) reducing the speed of the cross from 5°/s to 2.5°/s, (2) changing the axis of tracking from vertical to horizontal, or (3) employing a single inducer stimulus and a two interval forced choice procedure. In each case, no systematic difference between performance in in-phase and antiphase conditions was found.

Eccentricity

Phase-dependence of detection thresholds at the leading edge were remeasured at different eccentricities. This was achieved by displacing the target and inducer stimuli horizontally away from fixation. All other stimulus parameters were as described for the original phase-dependence experiment. As shown in Figure S3, although thresholds rise in absolute terms as the target stimulus is moved away from fixation, the magnitude of the difference between in-phase and antiphase conditions gradually decreases. This suggests an attenuation of the interference between sensory predictions and sensory input in the periphery.

Contrast-Defined Motion

As depicted in Figure S4A, we next repeated the phase-dependency experiment using contrast-defined motion. Target and inducer stimuli were generated by modulating the contrast of a full screen noise field (2 arcmin x 2 arcmin pixels, Michelson contrast = 0.5). Across a range of different conditions, we varied the nature of the noise carrier (static or dynamic) as well as the spatial frequency (1 c/°, 0.5 c/°) and temporal frequency (5 Hz, 2.5 Hz, 1.25 Hz) of the target/inducer contrast modulation. No systematic difference in modulation thresholds between in-phase and antiphase target conditions at the leading edge was found (see Figure S4B for example data set).

Supplemental Figure Legends

Figure S1, Related to Figure 1. Mean Differences in Log Contrast Detection Threshold between Leading- and Trailing-Edge Conditions, along with 95% Confidence Intervals

Thresholds at the leading edge are significantly better for in-phase targets (0, 2π), but significantly poorer for antiphase targets (π).

Figure S2, Related to Figure 2. Stimulus Configuration Used for Measuring Spatial Interference Patterns between Sensory Predictions and Sensory Input

Figure S3. No Predictive Modulation of Sensitivity for Purely Retinal Motion

(A) Example stimulus configuration, in which ocular tracking of the downward moving cross induces upward retinal motion in the inducer and target stimuli.

(B) Mean detection thresholds of three observers for in-phase (black) and antiphase (green) targets (upper panel). No difference in contrast sensitivity is evident (lower panel).

Figure S4. Eccentricity Dependence of Predictive Effect

Upper panel show mean detection thresholds of three observers for in-phase (black), antiphase (green) and baseline (unfilled) conditions, plotted as a function of target eccentricity. The magnitude of the phase-dependent effect is inversely related to target eccentricity (lower panel).

Figure S5.

(A) Example stimulus frame from contrast-defined motion experiment.

(B) Data obtained with 1 c/°, 5Hz modulations of a static noise carrier. No difference in mean detection thresholds ($n = 4$) was found between in-phase and antiphase targets presented at the leading edge of the inducer grating.

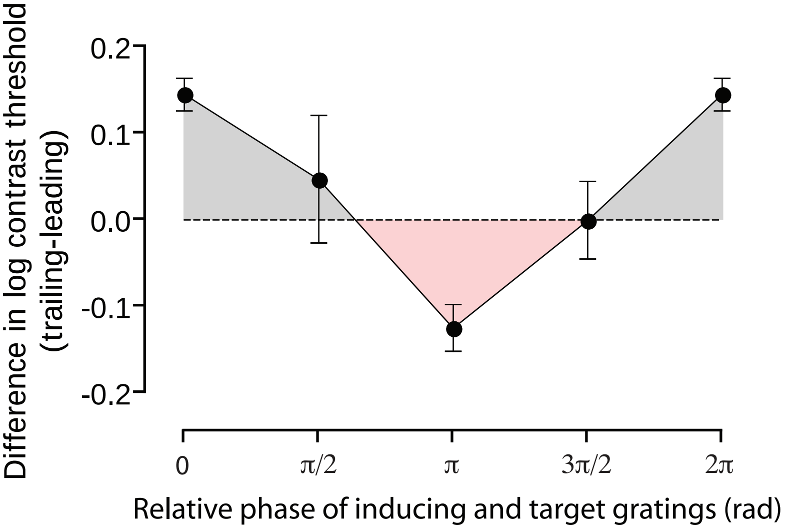

Difference in log contrast threshold
(trailing-leading)
0.2
0.1
0.0
-0.1
-0.2
0
π/2
π
3π/2
2π
Relative phase of inducing and target gratings (rad)

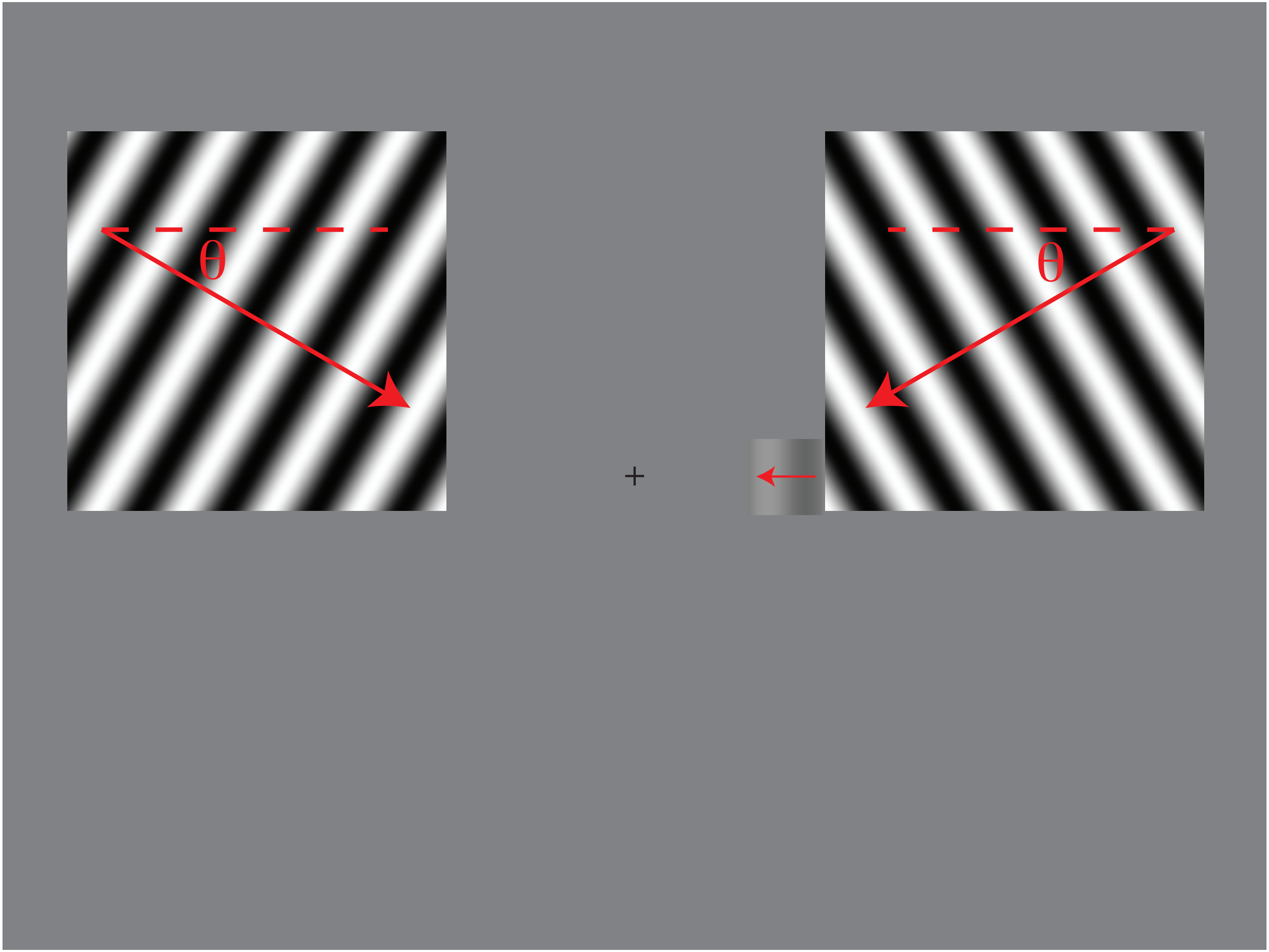
θ
θ

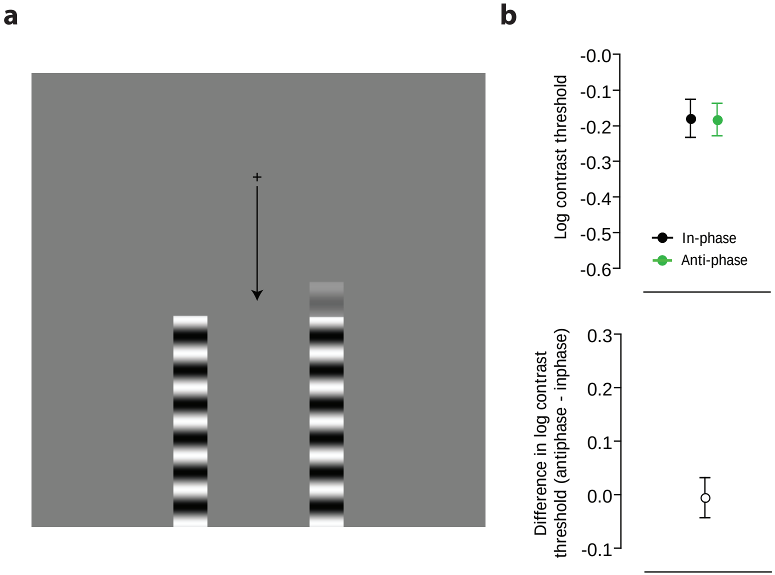

a
+
b
-0.0
-0.1
-0.2
-0.3
-0.4
-0.5
-0.6
Log contrast threshold
In-phase
Anti-phase
0.3
0.2
0.1
0.0
-0.1
Difference in log contrast threshold (antiphase - inphase)

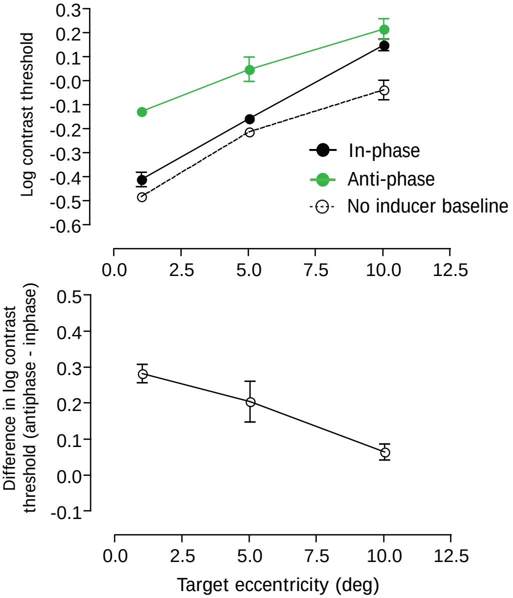

Log contrast threshold
0.3
0.2
0.1
-0.0
-0.1
-0.2
-0.3
-0.4
-0.5
-0.6
In-phase
Anti-phase
No inducer baseline
0.0
2.5
5.0
7.5
10.0
12.5
Difference in log contrast threshold (antiphase - inphase)
0.5
0.4
0.3
0.2
0.1
0.0
-0.1
0.0
2.5
5.0
7.5
10.0
12.5
Target eccentricity (deg)

a

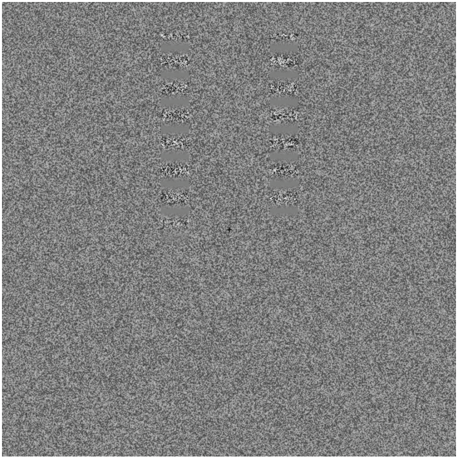

b

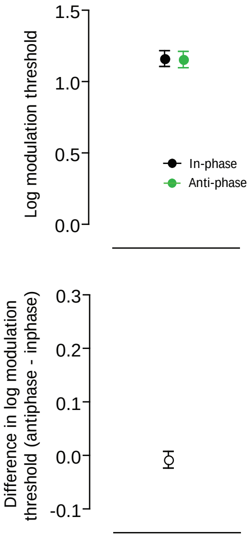
Log modulation threshold
1.5
1.0
0.5
0.0
In-phase
Anti-phase
Difference in log modulation threshold (antiphase - inphase)
0.3
0.2
0.1
0.0
-0.1
